# Design and validation of a wearable dynamometry system for knee extension-flexion torque measurement

**DOI:** 10.1038/s41598-024-60985-9

**Published:** 2024-05-07

**Authors:** Sungwoo Park, Youho Myong, Minwoo Cho, Seung Yeon Cho, Woo Hyung Lee, Byung-Mo Oh, Sungwan Kim

**Affiliations:** 1 Interdisciplinary Program in Bioengineering, The Graduate School, Seoul National University, 1 Gwanak-ro, Gwanak-gu, Seoul, 08826 Republic of Korea; 2 Innovative Medical Technology Research Institute, Seoul National University Hospital, 101 Daehak-ro, Jongno-gu, Seoul, 03080 Republic of Korea; 3 Department of Biomedical Engineering, Seoul National University College of Medicine, 103 Daehak-ro, Jongno-gu, Seoul, 03080 Republic of Korea; 4 Department of Rehabilitation Medicine, Seoul National University Hospital, Seoul National University College of Medicine, 101 Daehak-ro, Jongno-gu, Seoul, 03080 Republic of Korea; 5 Department of Transdisciplinary Medicine, Seoul National University Hospital, 101 Daehak-ro, Jongno gu, Seoul, 03080 Republic of Korea; 6 Institute on Aging, Seoul National University, 103 Daehak-ro, Jongno-gu, Seoul, 03080 Republic of Korea; 7 Institute of Bio engineering, Seoul National University, 1 Gwanak-ro, Gwanak-gu, Seoul, 08826 Republic of Korea

**Keywords:** Biomedical engineering, Rehabilitation

## Abstract

Muscle strength assessments are vital in rehabilitation, orthopedics, and sports medicine. However, current methods used in clinical settings, such as manual muscle testing and hand-held dynamometers, often lack reliability, and isokinetic dynamometers (IKD), while reliable, are not easily portable. The aim of this study was to design and validate a wearable dynamometry system with high accessibility, accuracy, and reliability, and to validate the device. Therefore, we designed a wearable dynamometry system (WDS) equipped with knee joint torque sensors. To validate this WDS, we measured knee extension and flexion strength in 39 healthy adults using both the IKD and WDS. Comparing maximal isometric torque measurements, WDS and IKD showed strong correlation and good reliability for extension (Pearson’s r: 0.900; intraclass correlation coefficient [ICC]: 0.893; standard error of measurement [SEM]: 9.85%; minimal detectable change [MDC]: 27.31%) and flexion (Pearson’s r: 0.870; ICC: 0.857; SEM: 11.93%; MDC: 33.07%). WDS demonstrated excellent inter-rater (Pearson’s r: 0.990; ICC: 0.993; SEM: 4.05%) and test–retest (Pearson’s r: 0.970; ICC: 0.984; SEM: 6.15%) reliability during extension/flexion. User feedback from 35 participants, including healthcare professionals, underscores WDS's positive user experience and clinical potential. The proposed WDS is a suitable alternative to IKD, providing high accuracy, reliability, and potentially greater accessibility.

## Introduction

Muscle strength is an important clinical outcome in rehabilitation, orthopedics, and sports medicine. Muscular weakness independently predicts all-cause mortality in healthy populations^1,2^. Furthermore, increased muscle strength in older adults has been associated with longer survival, improved functional outcomes, and enhanced quality of life^3^. In addition, reduced muscle strength has been identified as a contributing factor to cognitive decline and Alzheimer’s disease and seems to influence the long-term risk of stroke^4,5^. For muscle strength evaluation, the quadriceps and hamstrings, which are involved in knee extension and flexion, have been identified as effective muscle groups to assess declining muscle strength in a variety of chronic disorders^6,7^.

In clinical settings, the strength of the quadriceps and hamstrings is assessed using manual muscle tests (MMT) or handheld dynamometers (HHDs) that can quantitatively represent muscle strength^8^. However, MMT cannot provide a quantitative measure of muscle strength, making it difficult to objectively record changes in strength over time. In addition, MMTs and HHDs are susceptible to errors introduced by raters, which affect their accuracy and reliability^9^. In particular, when assessing powerful muscle groups, such as the quadriceps, challenges arise if the force to be measured exceeds the rater's weight and muscle strength. In such cases, the rater may be unable to stabilize the limbs and/or device, leading to difficulties obtaining accurate measurements^10,11^.

Isokinetic dynamometry (IKD) equipment, such as the Biodex System 4 Pro (Biodex Medical System Inc., Shirley, NY, USA), is considered the gold standard for measuring muscle strength and demonstrates high accuracy and reliability^12^. Despite the accuracy and reliability of IKDs, their limited portability and cost limit their use in regular hospital monitoring and athletic field evaluations^13,14^. Various muscle strength evaluation devices have been proposed to address the limitations of MMT, HHDs, and IKDs.

Sung et al. proposed an anchoring frame to assist in stabilizing HHD when measuring in the supine position^15^, and Hogrel et al. introduced a portable system for measuring knee extension in a seated position^16^. Padulo et al. designed an isometric bench that, although not highly portable, allowed the measurement of knee muscle strength in a seated position^17^. Compared with traditional methods, these inventions have the advantage of improving reliability and portability. However, they still present limitations, such as being bulky and inconvenient for transportation or requiring anchoring to bed frames, walls, or benches; therefore, they are influenced by environmental conditions^17–19^.

Myong et al.^20^ and Brookshaw et al.^21^ proposed devices that utilize load cells attached to the limb, allowing the measurement of knee extension, elbow extension, and elbow flexion strength without the need to install equipment in the surrounding environment. Cho et al.^22^ proposed a device that used load cells to measure the dorsiflexion and plantar flexion of the ankle. These devices demonstrated portability, validity, and reliability comparable to those of IKD. The portable articulated dynamometry system (PADS) proposed by Myong et al. is a device which facilitates the assessment of knee extension strength by securing load cells to the limbs, without requiring the installation or attachment of any equipment to the surrounding environment. This device functioned as a precursor to the current research^20^. Even though the PADS has enhanced accessibility for measuring maximum knee extension strength in daily life compared to the IKD, it has two primary limitations: Firstly, previous studies utilizing the PADS have only focused on assessing knee extension strength. As the hamstrings-to-quadriceps (H:Q) torque ratio is frequently used to detect muscle imbalances or as an indicator for injury prevention, prior studies have indicated that it is important to measure both knee extension and flexion strength together^23–25^. Secondly, in preliminary research using the PADS, the ability to adjust the length of the device based on limb length was restricted. Further, when measuring the force with a load cell to calculate the torque occurring at a knee joint, effort is required to measure the distance from the axis of rotation to the point of force application for each measurement. AS such, the PADS was intentionally designed without a feature for length adjustment.

In the present study, a wearable design for a muscle strength measurement system, in which torque sensors are positioned on the knee joint at the axis of rotation, is proposed. This study serves as a follow-up to previous PADS research using load cells and aims to improve the fixation force on the limb using a wearable design^20,26^. With the enhancement of fixation force, simultaneously measuring knee extension and flexion has become easier. The measurement method using torque sensors minimizes the distortion in the measurement values, even with deformations in the limb fixation and frame, and the symmetric design allows the intuitive setting of joint angles for measurement. In addition, the fixation is adjustable in length, enabling placement on high-stiffness areas with low-sensitive tissue, reducing pain or discomfort during measurement, and facilitating the measurement of maximum muscle strength^27^. The aim of this study was to design a wearable dynamometry system (WDS) and to evaluate its accuracy and reliability through cross-validation with measurements taken using the Biodex System 4 Pro in healthy adult subjects. Further, we report the results of a user experience questionnaire survey (UEQ-S).

## Results

In this study, the inter-rater and test–retest reliabilities of knee flexion and extension strength measurements were assessed in 39 healthy adults. Each participant underwent two individual evaluation sessions for intersession reliability conducted by two independent raters for both knees, resulting in 312 measurements for extension and flexion for each device (a wearable dynamometry system [WDS] and the IKD).

**Figure 1:**
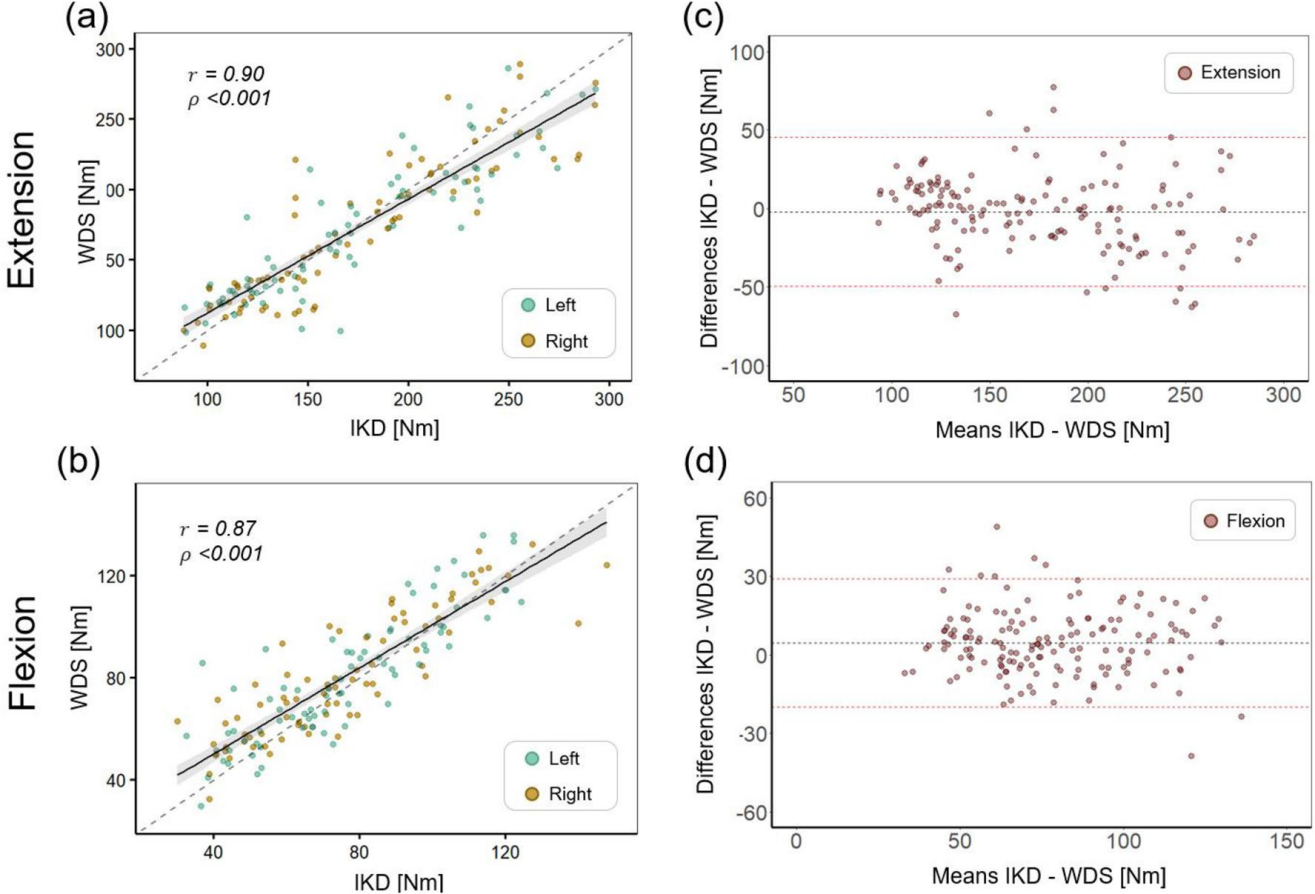
Linear regression analyses of the agreement between the wearable dynamometry system (WDS) and isokinetic dynamometry (IKD) for (**a**) knee extension measurements and (**b**) flexion measurements; Bland–Altman analyses of the agreement between the WDS and IKD for (**c**) knee extension measurements and (**d**) flexion measurements. The dotted line indicates the identity line, whereas the solid line indicates the linear regression line.

The cross-validation results for the WDS and IKD are presented in Table 1. The maximum extension and flexion torques measured using the WDS and IKD showed a strong correlation (Pearson’s r: 0.900 in extension; Pearson’s r: 0.870 in flexion). The maximal strength measured with the WDS and IKD revealed no statistically significant differences in both extension (mean difference: − 2.00 Nm; 95% CI − 13.75 to 9.74 Nm; *p*-value: 0.955) and flexion (mean difference: 4.54 Nm; 95% CI − 0.97 to 10.06 Nm; *p*-value: 0.758). The intraclass correlation coefficient (ICC) between the two devices was 0.893 for extension and 0.857 for flexion, indicating excellent reliability. The standard error of measurement (SEM) for extension and flexion was 17.12 Nm (9.85%) and 9.31 Nm (11.93%), respectively, and the minimal detectable change (MDC) at 95% confidence interval (CI) for extension and flexion was 47.47 Nm (27.31%) and 25.81 Nm (33.07%), respectively. The linear relationship and Bland–Altman analysis between the measurements taken with the two devices are demonstrated in Fig. 1. The measurements of extension and flexion strength by the two devices exhibited strong linearity (Fig. 1a and c). The mean difference between the extension strength measurements of the two devices was -2.00 Nm, with most differences falling within the 95% limits of agreement (LOA range: − 49.53 to 45.52 Nm) (Fig. 1c). The mean difference in flexion strength measurements between the two devices was 4.55 Nm, indicating a higher bias for the measurements using WDS; however, most differences remained within the 95% limits of agreement (LOA range: -19.99 to 29.08 Nm) (Fig. 1d).

**Table 1:** Summary of overall results: agreement, inter-rater reliability, and test–retest reliability of the WDS and IKD.

	Measurement (Nm)		CIM (Nm) [95% CI]	*p*-value^a^	Pearson’s *r*	ICC^b^	SEM (Nm)	MDC (Nm)
Agreement in extension								
WDS (ours)	174.80 ± 55.19		− 2.00 [− 13.75, 9.74]	0.955	0.900 (*p* < 0.001)	0.893	17.12 (9.85%)	47.47 (27.31%)
IKD (Biodex)	172.80 ± 49.68							
Agreement in flexion								
WDS (ours)	75.78 ± 25.04		4.54 [− 0.97, 10.06]	0.758	0.870 (*p* < 0.001)	0.857	9.31 (11.93%)	25.81 (33.07%)
IKD (Biodex)	80.33 ± 24.26							
Inter-rater reliability								
	Rater A	Rater B						
WDS (ours)	122.38 ± 58.75	123.59 ± 59.80	1.21 [− 8.13, 10.55]	0.972	0.990 (*p* < 0.001)	0.993	4.98 (4.05%)	13.79 (11.21%)
IKD (Biodex)	122.67 ± 62.31	123.01 ± 63.36	0.34 [− 9.56, 10.23]	0.993	0.980 (*p* < 0.001)	0.991	5.98 (4.86%)	16.56 (13.48%)
Test–retest reliability								
	Session I	Session II						
WDS (ours)	121.47 ± 57.98	124.50 ± 60.52	3.03 [− 6.30, 12.37]	0.937	0.970 (*p* < 0.001)	0.984	7.56 (6.15%)	20.96 (17.04%)
IKD (Biodex)	121.89 ± 62.52	123.79 ± 63.14	1.90 [− 8.00, 11.80]	0.964	0.970 (*p* < 0.001)	0.986	7.54 (6.13%)	20.89 (17.00%)

*CIM* change in mean, *CI* confidence interval, *ICC* intraclass correlation coefficient, *WDS* wearable dynamometry system, *IKD* isokinetic dynamometry, *SEM* standard error of measurement, *MDC* minimal detectable change.

^a^Permutation test was used to evaluate whether the difference in means between two measurements is statistically significant. ^b^ICC_(2,k)_ was used for agreement and inter-rater reliability, whereas ICC_(3,k)_ was used for test–retest reliability.

**Figure 2:**
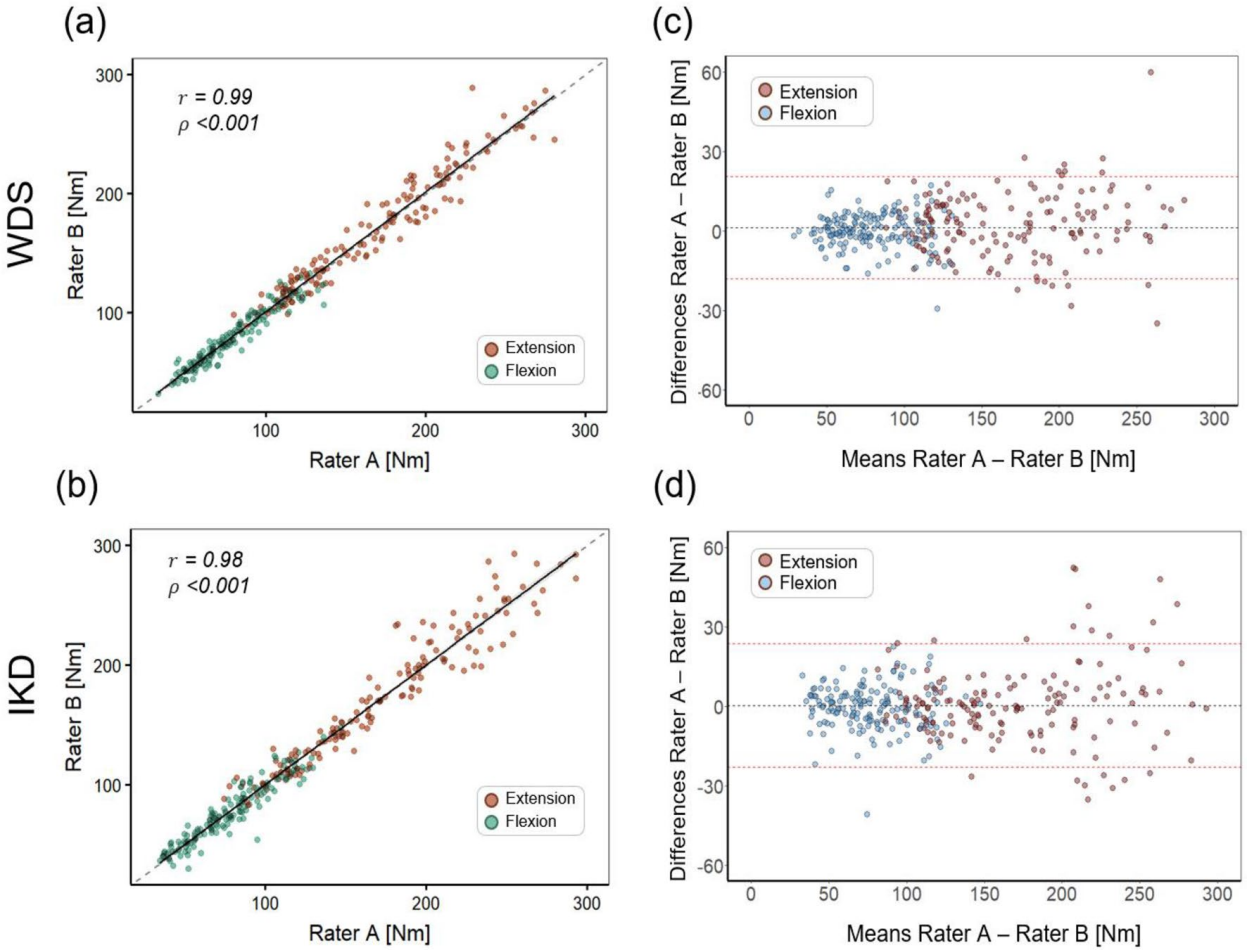
Linear regression analyses of the inter-rater reliability for the (**a**) wearable dynamometry system (WDS) and (**b**) isokinetic dynamometry (IKD); Bland–Altman analyses of the inter-rater reliability for the (**c**) WDS and (**d**) IKD.

The WDS was further validated by evaluating inter-rater reliability. Two independent raters assessed each participant, and the data obtained from each rater showed a strong interrater correlation (Pearson’s r: WDS, 0.990; IKD, 0.980) (Fig. 2). No significant inter-rater differences were observed for the WDS (mean difference: 1.21 Nm; 95% CI − 8.13, 10.55 Nm;* p*-value: 0.972) and IKD (mean difference: 0.34 Nm; 95% CI − 9.56, 10.23 Nm; *p*-value: 0.993). The ICC demonstrated excellent reliability for both the WDS and IKD (0.993 and 0.991, respectively). The mean SEM was 4.98 Nm (4.05%) for the WDS and 5.98 Nm (4.86%) for the IKD. The MDC was 13.79 Nm (11.21%) for the WDS and 16.56 Nm (13.48%) for the IKD (Table 1). The linear relationship and Bland–Altman analysis between the measurements of raters are shown in Fig. 2. Both the WDS and IKD showed strong linearity (Fig. 2a and c). The mean difference in measurements between raters using the WDS was 1.21 Nm, with the majority of differences falling within the 95% limits of agreement (LOA range: − 18.11 to 20.54 Nm) (Fig. 2c). The mean difference between the measurements of raters using the IKD was 0.34 Nm, with most differences also falling within the 95% limits of agreement (LOA range: − 23.01 to 23.68 Nm), although a higher occurrence of outliers was observed as the measurement values increased with the IKD (Fig. 2d).

**Figure 3:**
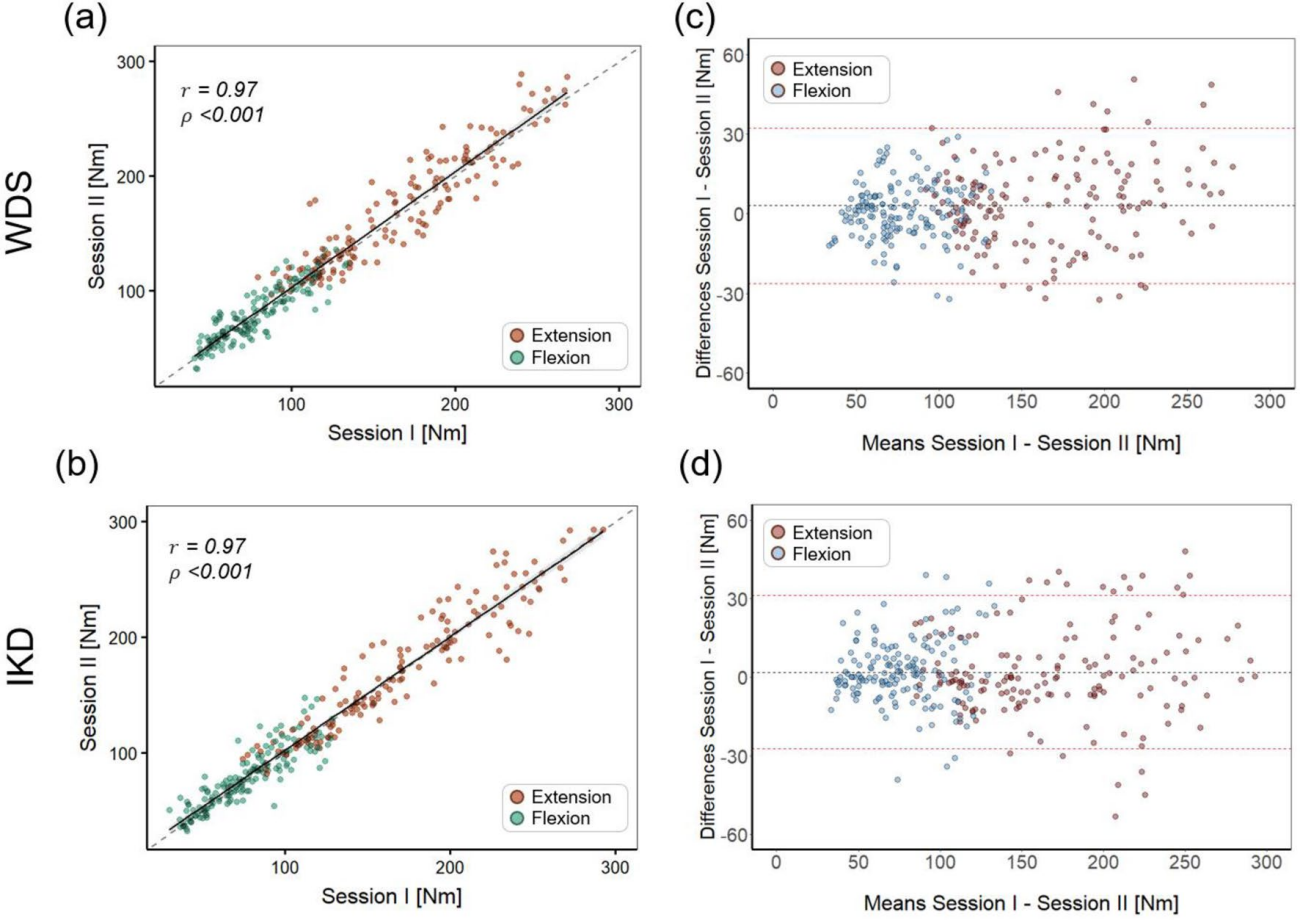
Linear regression analyses of the test–retest reliability for the (**a**) wearable dynamometry system (WDS) and (**b**) isokinetic dynamometry (IKD); Bland–Altman analyses of the test–retest reliability for the (**c**) WDS and (**d**) IKD.

All participants attended a second measurement session at least 24 h after the first session, and test–retest reliability was assessed by comparing the data from the two sessions. Data obtained from both sessions showed strong test–retest correlations (Pearson’s r: WDS, 0.970; IKD, 0.970) (Fig. 3). No significant differences were observed between the sessions for the WDS (mean difference: 3.03 Nm; 95% CI − 6.30, 12.37 Nm;* p*-value: 0.937) and IKD (mean difference: 1.90 Nm; 95% CI − 8.00, 11.80 Nm;* p*-value: 0.964). The ICC demonstrated excellent reliability for both the WDS and IKD (0.984 and 0.986, respectively). The mean SEM was 7.56 Nm (6.15%) for the WDS and 7.54 Nm (6.13%) for the IKD. The MDC was 20.96 Nm (17.04%) for the WDS and 20.89 Nm (17.00%) for the IKD (Table 1). The linear relationship and Bland–Altman analysis between session measurements are presented in Fig. 3. Both the WDS and IKD demonstrated strong linearity (Fig. 3a and c). The mean difference in session measurements using the WDS was 3.03 Nm, suggesting a potential learning effect from the test, with most differences within the 95% limits of agreement (LOA range: − 26.38 to 32.44 Nm) (Fig. 3c). The mean difference in session measurements using the IKD was 1.90 Nm, with most differences also within the 95% limits of agreement (LOA range: − 27.43 to 31.23 Nm) (Fig. 3d).

Of the 39 participants, 35 participated in a user experience survey using the UEQ-S. The UEQ-S results showed a significant difference in the overall user experience for the WDS (p < 0.001). Notably, the participants indicated that the WDS outperformed IKD in terms of ease of use, efficiency, clarity, inventiveness, and being on the leading edge. The results for each item are presented in Table 2.

**Table 2:** UEQ-S results (n = 35).

	IKD (Biodex)	WDS	CIM [95% CI]	*p*-value
Supportive	1.46 ± 1.20	1.94 ± 0.80	0.49 [0.24, 0.73]	0.051
Easy	**0.31 ± 1.98**	**2.14 ± 1.03**	**1.83 [1.45, 2.21]**	** < 0.001**
Efficient	**0.31 ± 1.75**	**1.94 ± 1.00**	**1.63 [1.29, 1.97]**	** < 0.001**
Clear	**0.46 ± 1.84**	**1.91 ± 0.95**	**1.46 [1.11, 1.81]**	** < 0.001**
Exciting	1.17 ± 1.10	1.46 ± 0.98	0.29 [0.04, 0.53]	0.255
Interesting	1.14 ± 1.06	1.57 ± 1.12	0.43 [0.17, 0.69]	0.105
Inventive	**0.31 ± 1.53**	**1.97 ± 0.92**	**1.66 [1.36, 1.96]**	** < 0.001**
Leading edge	**0.34 ± 1.51**	**1.74 ± 0.89**	**1.40 [1.10, 1.70]**	** < 0.001**
Total score	**5.51 ± 9.95**	**14.69 ± 6.30**	**9.17 [7.19, 11.20]**	** < 0.001**

*CIM* change in mean, *CI* confidence interval, *WDS* wearable dynamometry system, *IKD* isokinetic dynamometry.

Significant values are in bold.

## Discussion

The quadriceps and hamstrings play important roles, such as walking and balance, in daily life. Measuring knee extension and flexion strength is an effective method to assess the strength of the quadriceps and hamstrings. IKD, known as a benchmark for assessing muscle strength, is distinguished by its exceptional precision and reliability^12^. However, its high price, large dimensions (132 × 165 × 152 cm), and weight (612 kg) have restricted its widespread use in regular testing owing to its limited accessibility^14,15^ (see Supplementary Table S1 online). In addition, both MMT and HHDs are convenient for use in clinical settings and are commonly used for routine assessments; however, both methods are susceptible to errors introduced by raters^28,29^. In particular, measurement becomes challenging and unreliable if the force being assessed exceeds the rater's weight and muscular strength, causing the rater to be pushed back or unable to maintain the resistance^10,30^. Therefore, the research team recognized the need to develop a dynamometer system with good portability and high reliability.

The PADS previously developed by our research team was foldable and easy to carry and showed high validity and reliability in measuring the extension torque of the knee^20^. However, previously developed devices have limitations. First, for convenience in the torque calculation, the length of the lever arm in the PADS was fixed, making it impossible to adjust the fixation point to match the length of the leg. Regarding IKD, when measuring the maximum torque, the fixation point was positioned approximately 5 cm proximal to the distal aspect of the lateral malleolus to reduce discomfort and accurately transfer force^31^. However, with PADS, there was no adjustment for the fixation point's location, which occasionally caused discomfort or pain^20^. Second, the PADS had an aluminum frame in contact with the lower thigh, popliteal fossa, and calf. The fixation part was covered with silicone but was made of a rigid plastic material. Owing to the placement of the rigid material in the direction of the applied force, forces > 300 Nm led to pain caused by pressure from the fixation part^32^. In addition, there were cases in which the frame's contact with the patient's thigh or popliteal fossa caused discomfort.

In the present study, an ergonomically designed device was developed to measure the maximum extension and flexion torques of the knee. The proposed device is designed with an exoskeleton structure in which a torque sensor is placed concentrically on the knee joint, and a rigid frame acting as a lever arm is located on the left and right sides of the limb to surround the limb. This arrangement ensures that when assessing the force exerted by the knee joint, the participants can apply pressure without experiencing significant constriction or impediment from structural elements such as a frame. In addition, PADS using the load cell made it challenging to adjust the position of the fixation point because of the importance of the lever arm length for torque calculation. However, by incorporating the torque sensor, adjusting the fixation point to fit the leg length became more straightforward. This facilitates the attainment of maximal isometric extension and flexion torque, resulting in accurate and reliable measurements by the device^33,34^.

The results of the present study showed that the WDS demonstrated good agreement with the IKD in measuring knee isometric extension and flexion torque. In a study that used a load cell to measure knee isometric extension torque using PADS, the inter-rater SEM was 5.51%^20^. The WDS proposed in the present study showed an inter-rater SEM of 3.72%, which is comparable to previous results^20^. In addition, the intersession SEM for PADS was 8.38% in extension^20^, and that for WDS in the present study was 5.47% in extension, which is comparable to previous results (see Supplementary Table S2 online).

The ICC of the device proposed by Hogrel et al. and IKD was 0.973, and the SEM showed a good agreement of approximately 12%^16^. In addition, the ICC of the device proposed by Myong et al. and IKD was 0.935, and the SEM showed a good agreement of 9.75%^20^. For knee extension, the outcomes of the WDS proposed in the present study were comparable to those of existing devices, with an ICC of 0.893 and SEM of 9.85% for extension and an ICC of 0.857 and SEM of 11.93% for flexion.

In a user experience study, respondents expressed that the WDS was easier to use, more efficient, clearer, more innovative, and had a greater leading edge than the IKD. Approximately 25% of the participants (n = 9) were healthcare professionals such as doctors, nurses, physical therapists, and occupational therapists working at a university hospital. Healthcare professionals evaluated the system as easy to use, efficient, and clear (see Supplementary Table S3 online). Positive evaluations from these individuals are expected to play a significant role in the subsequent implementation of the system in a clinical setting.

The WDS is a wearable device that allows patients to measure maximal isometric knee extension and flexion torque in various positions. Weighing approximately 2.85 kg, it is portable and features an intuitive design with a high-precision torque sensor positioned at the axis of the knee joint rotation that exhibits low crosstalk^35^. To minimize bending or distortion, a stainless steel frame was used to withstand high forces. The WDS has yet to be commercialized; however, patents for WDS are currently being processed through the PCT system for international patents. In addition, the WDS is being prepared for product certification by obtaining Korean good manufacturing practice approval, making it easily accessible to researchers, healthcare professionals, and general users.

This study has some limitations; therefore, the interpretation of its findings should be approached with caution. First, because only healthy young adults aged 20–39 years were enrolled, the proposed device was not verified for individuals with relatively weak quadriceps and hamstrings, such as patients with musculoskeletal disorders or older adults. Additionally, this system has not yet been tested on stronger individuals, or those who have undergone strength training, such as athletes. Therefore, further research targeting patient populations or specifically focusing on older adults and athletes could validate the device in the future.

Second, when measuring knee extension and flexion torque using the WDS developed in this study, a learning effect may be observed. Although participants received training and performed practice attempts prior to undergoing testing, a learning effect could have been reflected in the measurements. Indeed, the change in the mean between sessions for both WDS and IKD was greater than zero, indicating an increasing trend for both devices.

Third, the device proposed in this study was developed as an isometric measurement system primarily aimed at enhancing portability. In addition to isometric methods, typical isokinetic and isotonic methods are used for measuring muscle strength. Isometric methods measure muscle strength while the joint angle is fixed, whereas isokinetic and isotonic methods measure muscle strength while moving the joint angle. Because movement speed is also an important factor in evaluating daily living performance, it should be considered when measuring muscle strength^36^. Isokinetic testing is extensively used to assess muscle strength while minimizing the risk of muscle injury^37^. However, applying it to portable devices is not easy because a driving system that includes an actuator is essential for isokinetic measurements. Therefore, our research team aims to conduct future research to develop a module that includes not only a torque sensor but also an actuator. Therefore, we expect to expand the application range of this device to isokinetic measurements and passive motion in knee rehabilitation.

In conclusion, the precise measurement of muscle strength and tracking of its trends are important in a clinical setting. However, conventional methods for assessing muscle strength, such as MMT and HHDs with limited reliability and reproducibility or the less accessible IKD, have been the primary approaches. In this study, the WDS demonstrated substantial reliability in measuring knee extension and flexion in healthy adults, with good accessibility and cost efficiency. The proposed WDS is an efficient alternative to established MMT and IKD.

## Methods

### Participants

This study included 39 healthy adults. Pregnant patients, those with a recent history of cardiovascular, musculoskeletal, neurological, or infectious diseases, and those who could not provide consent were excluded. In the study, the participants were Koreans aged between 20 and 39 years. Measurements of height, weight, and maximal grip strength were collected for all participants to evaluate physical characteristics (see Supplementary Table S4 online). This study followed the Declaration of Helsinki and was approved by the Institutional Review Board of Seoul National University Hospital (IRB no. 2201-025-1288). Written informed consent was obtained from all participants. The participant appearing in Fig. 6 provided written informed consent to publish the figure in an online open-access publication.

### Sampling size and methods

Based on previous research data^20^, a minimum of 16 participants were required. This calculation was performed to determine the sample size necessary to observe a maximum 95% limit of agreement (LOA) in Bland–Altman analysis^38^. The sample size was calculated with a statistical power of 90% and a significance level of 0.05 (two-tailed). Additionally, the convenience sampling method was applied to recruit participants who were easily accessible within the research setting and conformed to the study criteria.

### WDS

The WDS is a portable device featuring an improved exoskeleton design applied to the PADS^20^. The exoskeleton design facilitates joint angle adjustment and allows the measurement of extension and flexion forces in the same posture by aligning the knee joint and torque sensor axes^39^.

The main frame was composed of stainless steel (SUS304) with a thickness of 5 mm, which provided sufficient rigidity for muscle strength measurements. The design model used in the present study enabled angle adjustment at 5° intervals based on the combination of the main frame and the torque sensor.

The main frame was connected to an adjustable fixture tailored to the leg length and featured pads made of cotton and silicone (Ecoflex; Smooth-On, Inc., Macungie, PA, USA). It was designed to be secured to the thigh and calf using Velcro belts.

**Figure 4:**
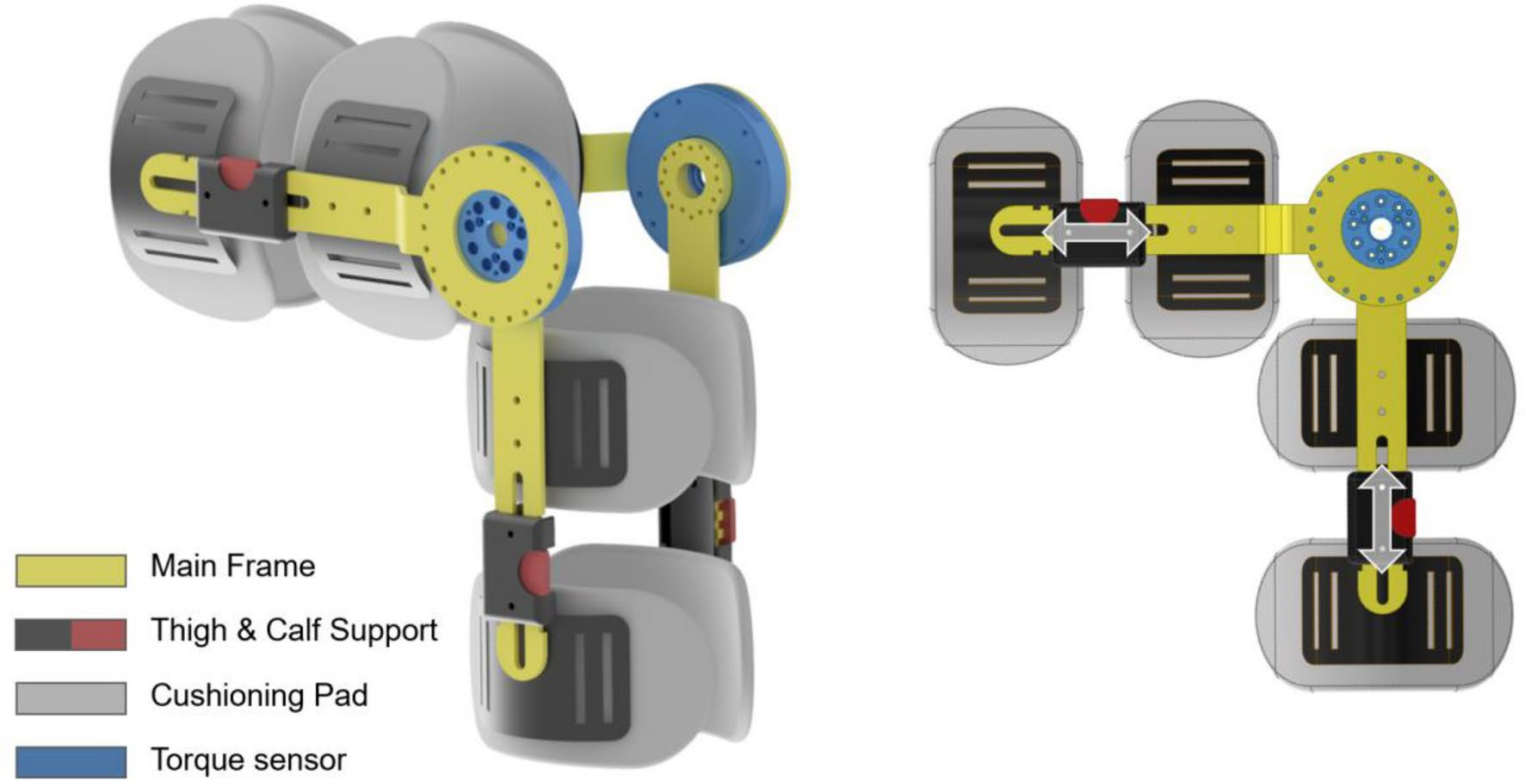
Component configuration and design features of the wearable dynamometry system.

**Figure 5:**
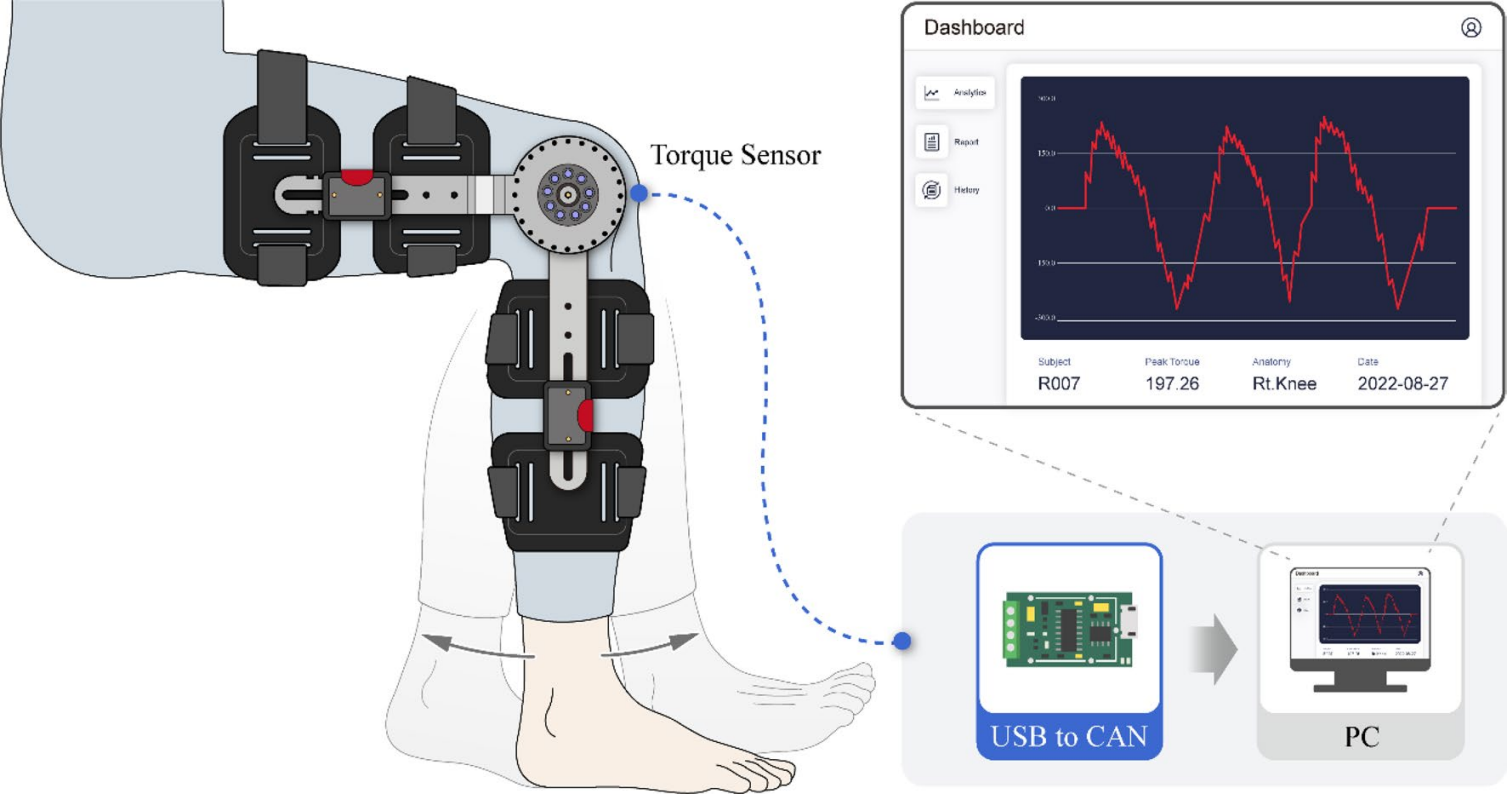
Conceptual diagram and visual representation of measurement using the device.

The torque sensor, which is responsible for measuring the rotational force of the knee joint, was positioned on the same axis as the rotational axis of the knee joint to ensure accurate torque measurement. In this study, a knee joint torque measurement system was used with two torque sensors (ATS200-D100; Aidin Robotics, Gyeonggi-do, South Korea) positioned bilaterally (Fig. 4). Each sensor could measure the torque within the range of 0–200 Nm with a resolution of 0.25 Nm. The sensor is thin and lightweight and effectively minimizes crosstalk using an Artificial Neural Network-based calibration method to obtain torque values excluding the moment^35,40^. The signal outputs from both sensors were transmitted to a laptop for data visualization and recorded through a universal serial bus to a controller area network transceiver (Fig. 5).

Validation was performed using a Class M1 weight set ranging from 0.1 to 100 kg within the measurement range (see Supplementary Fig. S1 online).

### Experimental design

**Figure 6:**
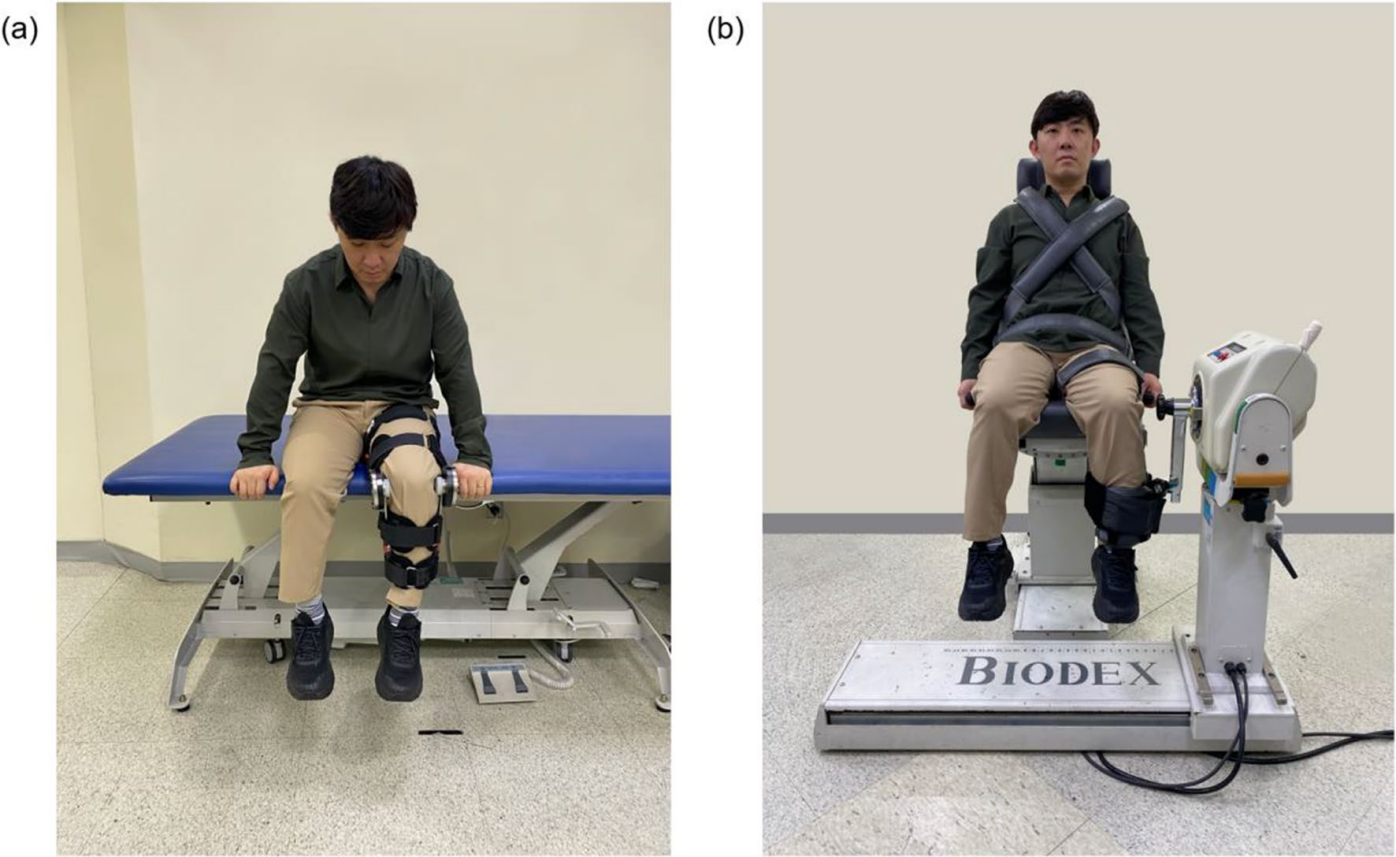
Device wearing configuration for a clinical trial of knee muscle strength measurement: (**a**) the wearable dynamometry system and (**b**) Isometric setup of the isokinetic dynamometry.

Maximal isometric extension and flexion torques of the knee joint were measured at 90° using the isometric setup of the WDS and IKD (Biodex System 4 Pro; Biodex Medical Systems Inc., Shirley, NY, USA) (Fig. 6). We aimed to evaluate the test–retest reliability of the two devices through two measurements (sessions) at least 24 h apart for each participant. In each session, measurements for both knees were carried out by two independent raters. A single trial consisted of extension and flexion movements, each lasting 3 s. Between each trial, a 30-s rest period was provided, while each rater recorded the maximum value measured across the two trials as the measurement outcome. When switching raters or changing the device, participants were allowed a 3-min rest period (see Supplementary Fig. S2 online). One rater was a board-certified physiatrist with 6 years of clinical experience, whereas the other was an engineer who was part of the development team. Before each trial, the participants were instructed to exert maximal effort and were provided consistent verbal encouragement throughout the trial. All participants performed a 3-min stretching session with the physiatrist before each assessment, and all assessments were performed under the direct supervision of the same physiatrist.

### User experience study

After all measurements were completed, participants were asked to fill out the UEQ-S form for both devices. The UEQ-S is a simple evaluation method that measures users' subjective perceptions of a product, such as attractiveness and efficiency. Each item in the UEQ-S is rated on a 7-point Likert scale ranging from − 3 (very negative) to + 3 (very positive)^41^. All participants completed the questionnaire voluntarily and anonymously.

### Data analysis

Due to the difference in the leg fixing positions between the WDS and the IKD, which resulted in different lever arm lengths, a constant corresponding to the ratio of the lever arm lengths was applied to the WDS to allow an absolute comparison of the measurements acquired using both devices. The constant value was 25/16 and was applied after the device calibration phase. The Kolmogorov–Smirnov test was applied to assess data normality, and a permutation test was performed to further assess whether there were any significant differences in measurements between the two devices, between raters, or between sessions. The inter-rater and test–retest reliabilities of each device were confirmed to assess the reliability of the WDS and IKD. The validity of the WDS was verified by considering IKD measurements as the gold standard and by comparing the values of the two devices^42^. The ICC established by Shrout and Fleiss was used to confirm the inter-rater and test–retest reliabilities and evaluate validity^43^. ICC model 2 (a two-way random effects model) was used to assess both the agreement between the two devices and the inter-rater reliability, whereas ICC model 3 (a two-way mixed effects model) was used to evaluate the test–retest reliability. The SEM was computed using the standard deviation (SD), where $SEM = SD \sqrt{1-ICC}$. Furthermore, the MDC at a 95% CI was determined using the formula: $MDC=1.96\cdot \sqrt{2}\cdot SEM$. To examine the inter-rater and test–retest reliabilities and the inconsistency of the values measured by the two devices, a Bland–Altman plot was drawn, and a scatter plot and Pearson’s correlation were shown for the two measured values^44^. All analyses were performed using R version 4.2.3.

### Supplementary Information

Supplementary Information.

## Data Availability

The data acquired in this study are not openly available due to the sensitive nature of human data (e.g. age, sex, height and weight). A de-identified dataset containing the full demographic and clinimetric data is available from the corresponding author (S.K.) upon reasonable request.
