# Supplementary material for: Design and validation of a wearable dynamometry system for knee extension-flexion torque measurement
Source: Sci Rep. 2024 May 7;14:10428. doi: 10.1038/s41598-024-60985-9 (PMC11076506; doi:10.1038/s41598-024-60985-9)
Supplement: Supplementary file 1 — Supplementary Information. [file 41598_2024_60985_MOESM1_ESM.docx]

**Supplementary Table S1**. Comparison of specifications for the WDS and IKD

|  | **Dimensions(cm)** | **Mass(kg)** | **Sensor** | **Mode** |
| --- | --- | --- | --- | --- |
| **WDS** | 30 x 30 x 9 | 2.85 | Axially aligned torque  (700 Nm max) | Isometric |
| **IKD**  **(Biodex 4 pro)** | 132 x 165 x 152 | 612 | Axially aligned torque  (680 Nm max) | Isometric  Isokinetic resistance  Passive motion |

WDS, wearable dynamometry system; IKD, isokinetic dynamometry.

**Supplementary Table S2.** Inter-rater and Test-retest reliabilities of the WDS and IKD: Calculated separately for extension and flexion

|  | Measurement (Nm) | | CIM (Nm) [95% CI] | *p*-value ^a)^ | Pearson’s *r* | | | ICC ^b)^ | SEM (Nm) | | MDC (Nm) |  |
| --- | --- | --- | --- | --- | --- | --- | --- | --- | --- | --- | --- | --- |
| Inter-rater Reliability | | | | | | | | | | | | |
| Extension | Rater A | Rater B |  | | | | | | | | | |
| WDS (ours) | 166.92 ± 48.59 | 169.24 ± 49.29 | 2.32 [-8.63, 13.26] | 0.938 | 0.970  (*p* <0.001) | | | 0.984 | 6.26 (3.72%) | | 17.34 (10.32%) |  |
| IKD (Biodex) | 169.17 ± 52.82 | 170.23 ± 54.21 | 1.06 [-10.91, 13.03] | 0.975 | 0.960  (*p* <0.001) | | | 0.981 | 7.33 (4.32%) | | 20.33 (11.98%) |  |
| Flexion |  |  |  |  |  |  |  | | |  |  |  |
| WDS (ours) | 77.84 ± 23.89 | 77.94 ± 23.50 | 0.10 [-5.20, 5.40] | 0.993 | 0.960  (*p* <0.001) | | | 0.981 | 3.29 (4.23%) | | 9.13 (11.72%) |  |
| IKD (Biodex) | 76.17 ± 25.45 | 75.78 ± 25.04 | -0.39 [-6.04, 5.26] | 0.979 | 0.940  (*p* <0.001) | | | 0.971 | 4.30 (5.66%) | | 11.93 (15.70%) |  |
| Test-retest Reliability | | | | | | | | | | | | |
| Extension | Session Ⅰ | Session Ⅱ |  | | | | | | | | | |
| WDS (ours) | 165.66 ± 47.69 | 170.49 ± 50.05 | 4.83 [-6.10, 15.77] | 0.872 | 0.930  (*p* <0.001) | | | 0.965 | 9.20 (5.47%) | | 25.49 (15.17%) |  |
| IKD (Biodex) | 168.91 ± 52.79 | 170.50 ± 54.24 | 1.59 [-10.38, 13.56] | 0.961 | 0.950  (*p* <0.001) | | | 0.974 | 8.69 (5.12%) | | 24.09 (14.19%) |  |
| Flexion |  |  |  |  |  |  |  | | |  |  |  |
| WDS (ours) | 77.27 ± 23.21 | 78.51 ± 24.16 | 1.23 [-4.07, 6.53] | 0.933 | 0.890  (*p* <0.001) | | | 0.944 | 5.59 (7.17%) | | 15.49 (19.88%) |  |
| IKD (Biodex) | 74.87 ± 24.62 | 77.08 ± 25.81 | 2.21 [-3.43, 7.85] | 0.879 | 0.880  (*p* <0.001) | | | 0.935 | 6.42 (8.45%) | | 17.79 (23.41%) |  |

CIM, change in mean; CI, confidence interval; ICC, intraclass correlation coefficient; WDS, wearable dynamometry system; IKD, isokinetic dynamometry; SEM, standard error of measurement; MDC, minimal detectable change. ^a)^permutation test was used to evaluate whether the difference in means between two measurements is statistically significant. ^b)^ICC_(2,k)_ was used for agreement and inter-rater reliability, whereas ICC_(3,k)_ was used for test-retest reliability.

**Supplementary Table S3.** UEQ-S results for healthcare professionals (n=9)

|  | **IKD** | **WDS** | **CIM [95% CI]** | ***p*-value** |
| --- | --- | --- | --- | --- |
| Supportive | 1.67±1.12 | 2.22±0.44 | 0.49 [0.24, 0.73] | 0.051 |
| **Easy** | **-0.11±1.96** | **2.56±0.53** | **2.67 [1.95, 3.39]** | **0.003** |
| **Efficient** | **0.11±1.69** | **2.11±0.93** | **2.00 [1.32, 2.68]** | **0.009** |
| **Clear** | **0.22±1.86** | **2.33±0.71** | **2.11 [1.41, 2.81]** | **0.009** |
| Exciting | 1.44±1.01 | 1.78±0.83 | 0.33 [-0.13, 0.80] | 0.458 |
| Interesting | 1.22±1.09 | 2.22±0.97 | 1.00 [0.48, 1.52] | 0.057 |
| Inventive | 0.56±1.59 | 2.11±0.78 | 1.56 [0.93, 2.18] | 0.022 |
| Leading edge | 0.67±1.50 | 1.70±0.82 | 1.03 [0.45, 1.62] | 0.091 |
| **Total score** | **5.78±8.84** | **17.10±4.08** | **11.30 [7.89, 14.80]** | **0.005** |

CIM, change in mean; CI, confidence interval; WDS, wearable dynamometry system; IKD, isokinetic dynamometry.

**Supplementary Table S4.** Demographics and anthropometry of study participants (n=39)

|  | Male (n = 17) | Female (n = 22) |
| --- | --- | --- |
| Age Group [years] | | |
| 20 – 29 | 21 (53.8%) | |
| 30 – 39 | 18 (46.2%) | |
| Age [years] | 30.12 $\pm$ 3.81 | 29.94 $\pm$ 4.05 |
| Height [cm] | 173.37 $\pm$ 4.62 | 162.88 $\pm$ 5.15 |
| Weight [kg] | 73.83 $\pm$ 9.22 | 54.15 $\pm$ 6.62 |
| Maximal Grip Strength [kg] | | |
| Right | 42.03 $\pm$ 6.57 | 28.09 $\pm$ 3.95 |
| Left | 41.98 $\pm$ 6.81 | 27.21 $\pm$ 4.31 |

cm, centimeter; kg, kilogram.


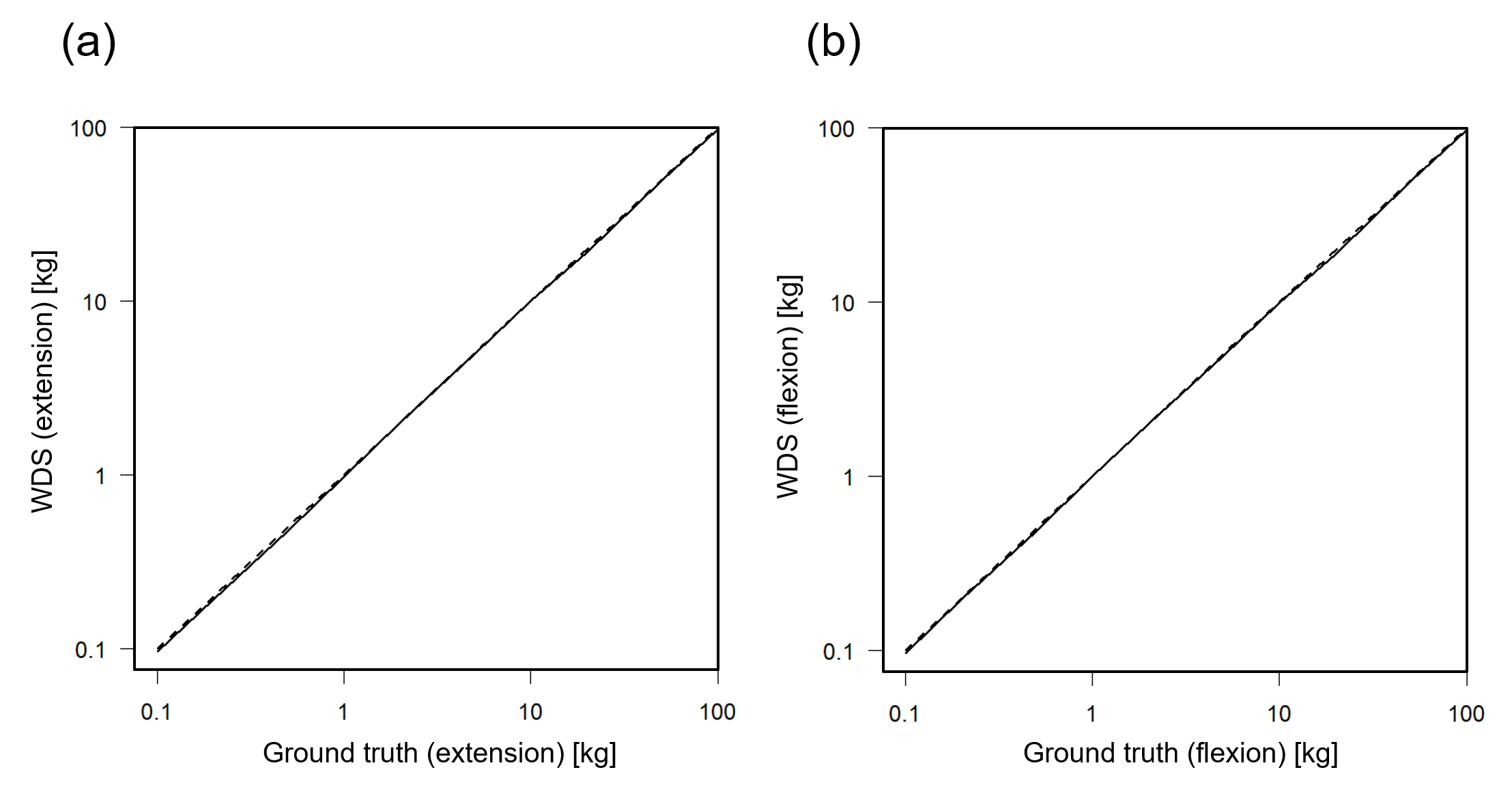


**Supplementary Fig. S1.** Calibration Curve of the WDS. The dotted line indicates the identity line, whereas the solid line indicates the calibration curve.


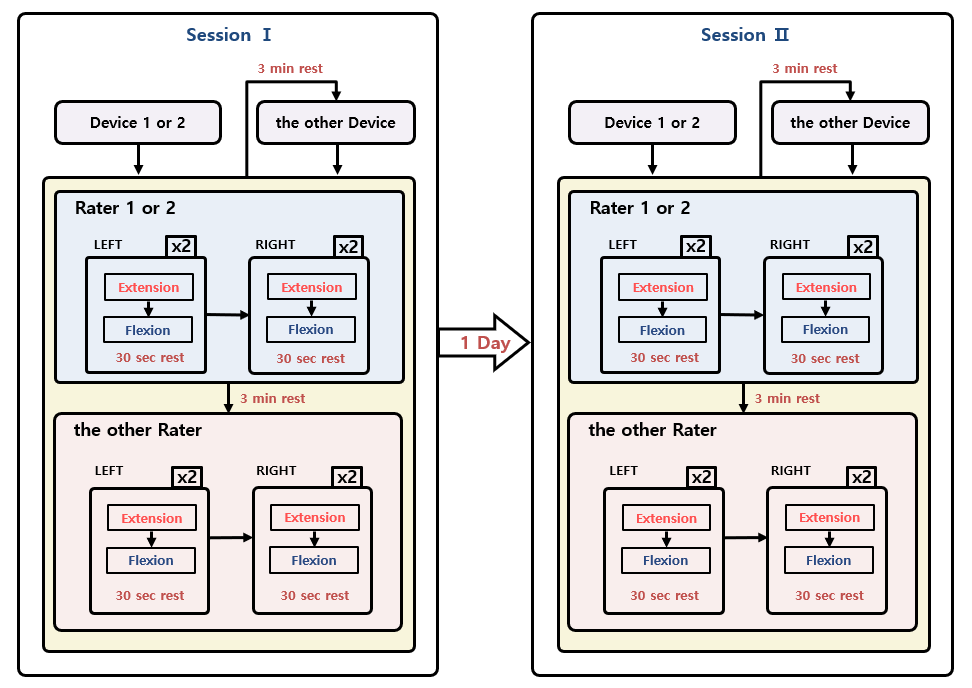


**Supplementary Fig. S2.** Flowchart representation of the overall protocols for strength assessment.
